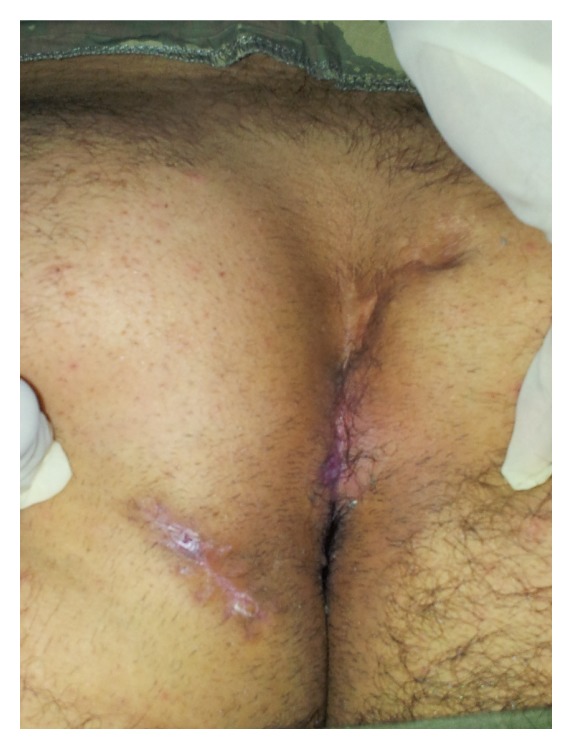# Minimally Invasive Surgical Approach to Complicated Recurrent Pilonidal Sinus

**DOI:** 10.1155/2015/759316

**Published:** 2015-10-21

**Authors:** Vahit Onur Gul, Sebahattin Destek, Serhat Ozer, Ergin Etkin, Serkan Ahioglu, Mehmet Ince, Vedat Cimin, Deniz Sen, Yesim Erbil

**Affiliations:** ^1^General Surgery Department, Edremit Military Hospital, Edremit, 10300 Balıkesir, Turkey; ^2^General Surgery Department, Tuzla Hospital, Tuzla, 34944 İstanbul, Turkey; ^3^General Surgery Department, Konya Military Hospital, 42040 Konya, Turkey; ^4^General Surgery Department, Gulhane Military Medical Faculty, 06180 Ankara, Turkey; ^5^General Surgery Department, Istanbul University Medical Faculty, Capa, 34093 Istanbul, Turkey

## Abstract

Pilonidal sinus is considered as a simple and frequently occurring disease localized at the sacrococcygeal area. However, at the intergluteal region, it can often turn into a chronic and complicated disease. In some cases, it can fistulize up to the gluteal region and appear at the secondary orifices. Minimally invasive surgical techniques are becoming widespread in recent years due to the increased experience and development of new instruments. Limited excision of the pilonidal sinus tract can be a better treatment option compared with large excisions in terms of recovery time and patient's comfort. This case study reports the single-phase surgical treatment of complicated and recurrent pilonidal sinus localized at the gluteal area, with minimal tissue loss and inflammation.

## 1. Introduction

Pilonidal sinus disease (PSD) is a chronic and inflammatory disease that often occurs at the sacrococcygeal region in men. Although the etiology is not exactly known, it is accepted that hair growth penetrating into the subcutaneous cysts results in foreign body reaction and infection [1]. The frequent complications of PSD are formation of cellulitis, abscess, and fistulae. Development of PSD necessitates surgical intervention, and there are several conservative and surgical treatments such as excision and primary closure, cryosurgery, marsupialization, and skin grafting. It is a common opinion that PSD should be treated with large excision and flap methods. Despite the availability of several techniques, the recurrent rates are still high, and the search for an ideal treatment is still ongoing [2, 3]. With new technical advancements in recent years, physicians are increasingly becoming attracted toward minimal surgical approaches for the treatment of chronic PSD.

## 2. Case Report

**Figure 1:**
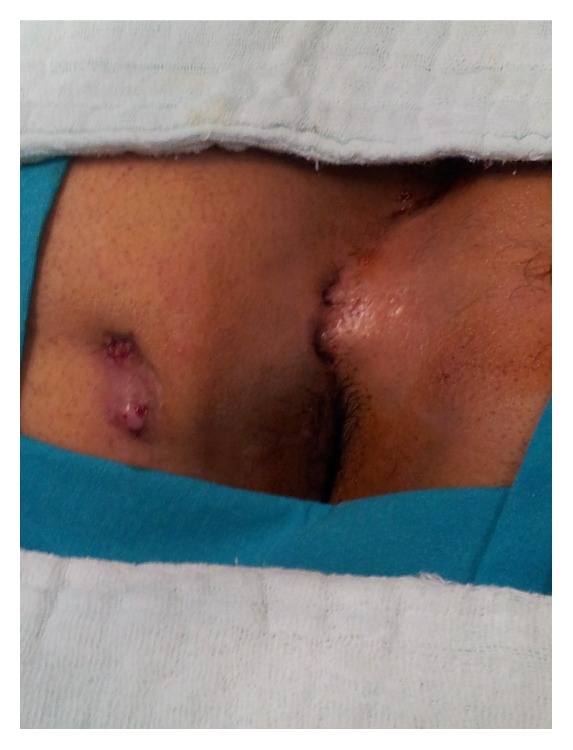


A 22-year-old male patient had undergone excision and primary closure surgery for PSD 2 years ago. The patient consulted our clinic approximately 6 months ago with complaints of pain initiating at the gluteal region and foul-smelling defluxion. Physical examination showed a scar tissue in the intergluteal line due to a forehand surgery, on which two recurrent pilonidal orifices at a distance of 8 cm from the anal canal and two secondary sinus orifices at a distance of 15 cm and seven o'clock alignment from these lesions were observed. Examination using a stylet confirmed that the orifices were related (Figure 1).

**Figure 2:**
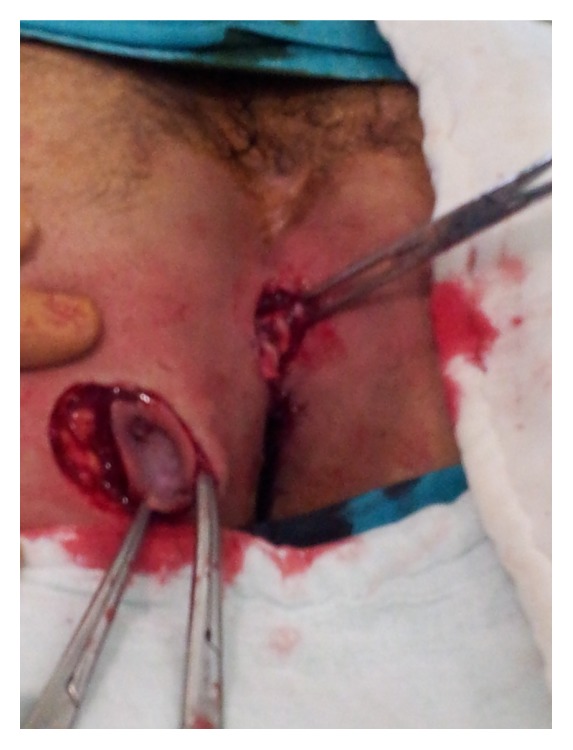


Two separate minimal ellipsoid cuts were made through the primary and secondary orifices so as to minimize tissue loss in the patient (Figure 2).

**Figure 3:**
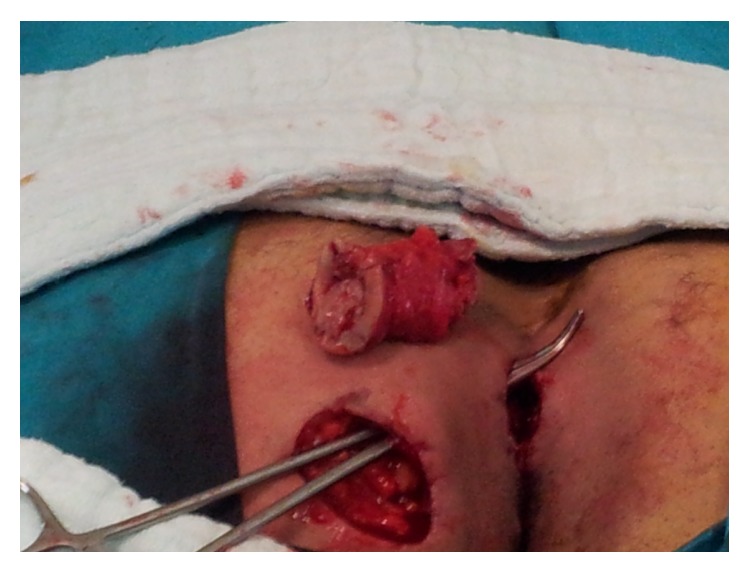


**Figure 4:**
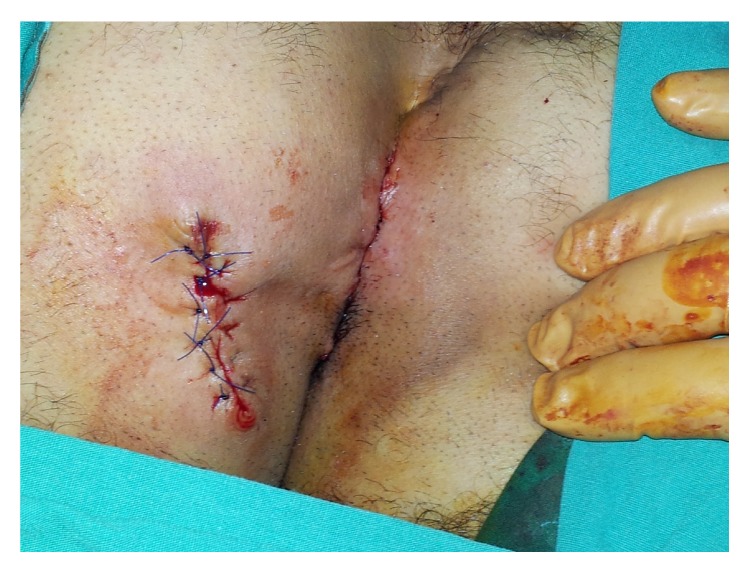


The fistula tract was unblocked by excision with obtuse and sharp dissections using a stylet, such that the subcutaneous tissue along with the tunnel-shaped sinus orifices and the healthy tissue remained between the two incisions (Figure 3). Subcutaneous fistulectomy was performed for closing the scar between the sides with primary recurrent PSD excision (Figure 4). Postoperative antibiotics were not administered to the patient and he was discharged on the second day postoperatively. The patient was followed up with intermittent examinations for 1 year. No complications including recurrent bleeding, formation of fistulae, and infection were observed during the follow-up period (Figure 5).

## 3. Discussion

PSD is a frequently occurring disease worldwide and can be diagnosed only by clinical findings and treated with various surgical methods. However, treatment of recurrent episodes and the nonhealing scars is difficult. There are various surgical techniques for its treatment, but all of them follow a common method, which is excision [4]. The most frequent complications occurring after PSD surgery are bleeding and infection. In the late period, recurrence and formation of fistulae are observed. Four important forms of the disease can be identified. These forms are directly related to the time gap between the beginning of the disease and the time that the patient seeks a clinic or any health care organization. The chronic fistulized form has been reported as being the most frequent by Doll et al. [5]. The aim of PSD treatment must be that the patients should resume their daily activities at the earliest, and the treatment has to be simple and of low cost, with low rates of recurrence and complications [2, 3, 6]. Minimally invasive surgical techniques are becoming more popular, consistent with the increasing experience and development of new instruments. Limited excision of the PSD tract has been shown to be a better treatment option with faster recovery and better patient compliance compared with large excisions [7]. Another study reported that the duration of hospitalization in case of the limited excision technique is 1.14 days, whereas flap techniques require 3.61 days [8]. Increased cosmetic expectations also increase the interest in minimal surgical practices. Large excisions cause limitations in the comfort of the patient's life. This could be more severe than the original disease itself, during the postoperative period. It is known that compared to classical surgical procedures, minimally invasive surgical approaches shorten the hospitalization and postoperative periods, provide high patient comfort with low cost, and help the patients resume their routine. Soll et al. performed limited excision (sinusectomy) and reported that the recurrent ratio is low and the patients were able to resume their normal activities in a shorter time [9]. In a randomized prospective study involving 83 patients, three different techniques, limited excision, secondary scar recovery, and primary closure, were compared. It was observed that although there were no significant differences between the three techniques, limited excision was found to be better in terms of shorter recovery period and patient satisfaction [10]. Gips et al. compared the long-term results of invasive surgical practice, which they performed using trephine, with those of other common surgical techniques in 1358 cases having PSD. They reported that the trephine group patients showed lower recurrence and morbidity rates compared to those treated with other classical techniques [11]. In our case, instead of a large excision, minimal excision with minimal tissue loss and inflammation was performed, and the obtained results are consistent with those of the abovementioned studies. We used a minimally invasive technique, that is, excision of the diseased area, instead of excision-primary closure and wide excision-flap techniques in recurrent and complicated cases. We closed the excised areas using primary closure. Although some studies indicate minimally invasive surgery for PSD, there are no studies describing the recurrent and complicated cases. Therefore, this case report could be helpful in reaching a consensus along with further studies on recurrent and complicated PSD cases.

## 4. Conclusion

Although several methods have been identified for PSD surgery, the debate about the best method is still ongoing. A minimally invasive approach is important for increasing the postoperative comfort of the patient, decreasing the loss of work, and decreasing the indirect costs. More studies are warranted in this area. In conclusion, we believe that execution of a minimally invasive surgical technique for PSD can be among the most important methods for treating not only primary PSD but also complicated and recurrent PSD cases.

## Figures and Tables

**Figure 5:**